# Supplementary material for: Burden of preconception morbidity in women of reproductive age from an urban setting in North India
Source: PLoS One. 2020 Jun 18;15(6):e0234768. doi: 10.1371/journal.pone.0234768 (PMC7302496; doi:10.1371/journal.pone.0234768)
Supplement: S1 Table — (DOCX) [file pone.0234768.s001.docx]

**Supplemental Table 1: Sample sizes**

|  | **Severe anemia** | **Mild to Moderate anemia** | **BMI <18.5 kg/m^2^** | **BMI <16 kg/m^2^** | **BMI ≥25 kg/m^2^** | **Hypothyroid** | **STIs/RTIs** |
| --- | --- | --- | --- | --- | --- | --- | --- |
| Expected Prevalence (%) | 3 | 51 | 16 | 3 | 25 | 7 | 40 |
| Expected 95% Confidence Interval | 2-4 | 45-55 | 13-19 | 2-4 | 21-29 | 5-9 | 36-44 |
| Relative Precision (%) | 30 | 10 | 20 | 30 | 15 | 30 | 10 |
| Required sample size | 1380 | 369 | 504 | 1380 | 512 | 567 | 576 |
| Total sample size including 20% missing specimens | 1656 | 443 | 605 | 1656 | 620 | 680 | 691 |

Severe anemia: hemoglobin level <8 g/dL; Mild to Moderate anemia: hemoglobin level 8 to 11.9 g/dL; BMI <18.5 kg/m^2^: undernutrition; BMI <16 kg/m^2^: severe undernutrition; BMI ≥25 kg/m^2^: overweight or obesity; Hypothyroid: TSH levels >5.5 IU/mL or if TSH levels between 4.0 and 5.5 IU/mL and FT4 levels were <0.89 ng/dL; STI/RTIs: presence of both symptoms and signs
