# Supplementary material for: Burden of preconception morbidity in women of reproductive age from an urban setting in North India
Source: PLoS One. 2020 Jun 18;15(6):e0234768. doi: 10.1371/journal.pone.0234768 (PMC7302496; doi:10.1371/journal.pone.0234768)
Supplement: S2 Table — (DOCX) [file pone.0234768.s002.docx]

**Supplemental Table 2: Predicted probabilities of baseline socio-demographic and anthropometry variables for Individual morbidities**

|  | **Margin (95% CI)** | | | | | |
| --- | --- | --- | --- | --- | --- | --- |
|  | **Moderate to Severe Anemia** | **Hypothyroidism** | **Undernutrition** | **Overweight or Obesity** | **Prediabetes and Diabetes** | **Symptoms and Signs of STI/RTI** |
| **Women’s age** | -0.006  (-0.013 to 0.001) | 0.01  (0.001 to 0.011) | -0.01  (-0.02 to -0.001) | 0.04  (0.03 to 0.05) | 0.01  (0.005 to 0.015) | 0.0004  (-0.004 to 0.005) |
| **Women’s years of schooling**  None (0)  Primary (1-5)  Secondary (6-12)  Higher than secondary (>12) | Ref  -0.05 (-0.17 to 0.05)  -0.12 (-0.22 to -0.02)  -0.19 (-0.29 to -0.08) | Ref  -0.06 (-0.15 to 0.03)  -0.08 (-0.16 to 0.004)  -0.06 (-0.15 to 0.02) | Ref  0.02 (-0.06 to 0.10)  -0.01 (-0.08 to 0.06)  -0.03 (-0.10 to 0.05) | Ref  0.03 (-0.08 to 0.13)  0.01 (-0.08 to 0.10)  0.02 (-0.07 to 0.11) | Ref  0.02 (-0.06 to 0.09)  0.01 (-0.06 to 0.07)  0.02 (-0.04 to 0.09) | Ref  0.07 (0.01 to 0.14)  0.04 (-0.01 to 0.09)  0.02 (-0.04 to 0.07) |
| **Women’s occupation**  Working  Housewife | Ref  -0.02 (-0.11 to 0.07) | Ref  -0.04 (-0.11 to 0.03) | Ref  -0.01 (-0.1 to 0.07) | Ref  -0.04 (-0.12 to 0.04) | Ref  -0.03 (-0.09 to 0.03) | Ref  -0.06 (-0.13 to 0.01) |
| **Religion of head of the household**  Others  Hindu | Ref  0.05 (0.001 to 0.10) | Ref  -0.03 (-0.07 to 0.01) | Ref  0.02 (-0.02 to 0.05) | Ref  -0.09 (-0.14 to -0.03) | Ref  0.001 (-0.03 to0.04) | Ref  -0.02 (-0.07 to 0.01) |
| **Wealth Quintiles**  Poorest  Very Poor  Poor  Less Poor  Least Poor | Ref  -0.01 (-0.07 to 0.04)  0.01 (-0.05 to 0.07)  -0.02 (-0.09 to 0.04)  -0.003 (-0.08 to 0.08) | Ref  0.03 (-0.01 to 0.08)  0.01 (-0.03 to 0.06)  0.02 (-0.03 to 0.07)  -0.002 (-0.06 to 0.05) | Ref  -0.06 (-0.11 to -0.01)  -0.07 (-0.12 to -0.01)  -0.10 (-0.16 to -0.04)  -0.12 (-0.18 to -0.05) | Ref  0.02 (-0.03 to 0.08)  0.03 (-0.03 to 0.09)  0.11 (0.04 to 0.17)  0.08 (0.005 to 0.16) | Ref  0.01 (-0.03 to 0.05)  0.02 (-0.02 to 0.07)  0.05 (0.004 to 0.09)  0.05 (-0.005 to 0.1) | Ref  -0.003 (-0.04 to.04)  0.004 (-0.04 to .05)  0.01 (-0.04 to 0.05)  0.02 (-0.04 to 0.08) |
| **Family structure**  Nuclear  Extended/Joint | Ref  -0.02 (-0.06 to 0.03) | Ref  0.004 (-0.03 to 0.04) | Ref  -0.01 (-0.04 to 0.03) | Ref  -0.03 (-0.07 to 0.01) | Ref  -0.01 (-0.04 to 0.02) | Ref  0.02 (-0.01 to 0.04) |
| **Women’s BMI**  18.5 to 24.99 kg/m^2^  <18.5 kg/m^2^  25 to 29.99 kg/m^2^  >= 30 kg/m^2^ | Ref  0.05 (-0.004 to 0.11)  -0.08 (-0.13 to -0.03)  -0.08 (-0.16 to 0.006) | Ref  -0.03 (-0.07 to 0.004)  -0.001 (-0.04 to 0.04)  0.01 (-0.06 to 0.07) |  |  | Ref  -0.02 (-0.04 to0.15)  0.09 (0.05 to 0.13)  0.34 (0.25 to 0.43) | Ref  0.06 (0.02 to 0.10)  0.01 (-0.03 to 0.04)  0.02 (-0.04 to 0.08) |
