# Supplementary material for: Burden of preconception morbidity in women of reproductive age from an urban setting in North India
Source: PLoS One. 2020 Jun 18;15(6):e0234768. doi: 10.1371/journal.pone.0234768 (PMC7302496; doi:10.1371/journal.pone.0234768)
Supplement: S3 Table — (DOCX) [file pone.0234768.s003.docx]

**Supplemental Table 3: Diagnostics of Multivariable Logistic Regression Models**

| **Models** | **Specification error** | **P value for Goodness-of-fit** | **Mean Variance inflation factor for Multicollinearity** | **Mean Pregibon leverage values for Influential observations** |
| --- | --- | --- | --- | --- |
| Moderate to Severe Anemia | _hat p value: 0.02 | 0.10 | 1.12 | Mean: 0.02  2 of 1957 observations have values >0.6 (>3 times of the average leverage) |
|  | _hatsq p value: 0.98 |  |  |  |
| Hypothyroidism | _hat p value: 0.02 | 0.60 | 1.12 | Mean: 0.02  0 of 1957 observations has values >0.6 (>3 times of the average leverage) |
|  | _hatsq p value: 0.57 |  |  |  |
| Undernutrition | _hat p value: 0.04 | 0.32 | 1.12 | Mean: 0.03  1 of 1963 observations has values >0.9 (>3 times of the average leverage) |
|  | _hatsq p value: 0.06 |  |  |  |
| Overweight or Obesity | _hat p value: 0.001 | 0.46 | 1.12 | Mean: 0.03  0 of 1963 observations has values >0.9 (>3 times of the average leverage) |
|  | _hatsq p value: 0.12 |  |  |  |
| Prediabetes or Diabetes | _hat p value: 0.001 | 0.77 | 1.12 | Mean: 0.02  5 of 1956 observations have values >0.6 (>3 times of the average leverage) |
|  | _hatsq p value: 0.22 |  |  |  |
| Symptoms and Signs of STIs/RTIs | _hat p value: 0.08 | 0.06 | 1.12 | Mean: 0.02  2 of 1963 observations have values >0.6 (>3 times of the average leverage) |
|  | _hatsq p value: 0.75 |  |  |  |

As this is a cross-sectional study, each observation is independent. The Stata command linktest was used to detect specification error. Linktest command uses the predicted value (_hat) and predicted value squared (_hatsq). The _hat should be statistically significant, since it is the predicted value from the model. On the other hand, if the model is properly specified, variable _hatsq shouldn’t have much predictive power except by chance. Supplemental Table 3 shows that _hat p values are significant and _hatsq are non-significant for all models. The p values of Hosmer and Lemeshow’s goodness-of-fit test are non-significant for all models. The mean Variance inflation factor (VIF) is 1.12 (close to 1) for all the models. It signifies that all of the variables are orthogonal to each other, in other words, completely uncorrelated with each other. There are very few observations where pregibon leverage value is more than 3 times of mean pregibon leverage value indicating very few influential observations.
