# Supplementary material for: Burden of preconception morbidity in women of reproductive age from an urban setting in North India
Source: PLoS One. 2020 Jun 18;15(6):e0234768. doi: 10.1371/journal.pone.0234768 (PMC7302496; doi:10.1371/journal.pone.0234768)
Supplement: Attachment — Submitted filename: Responses to reviewers.docx [file pone.0234768.s004.docx]

**RESPONSES TO REVIEWERS**

**1. We note that you have indicated that data from this study are available upon request. PLOS only allows data to be available upon request if there are legal or ethical restrictions on sharing data publicly. For information on unacceptable data access restrictions, please see** [**http://journals.plos.org/plosone/s/data-availability#loc-unacceptable-data-access-restrictions**](http://journals.plos.org/plosone/s/data-availability#loc-unacceptable-data-access-restrictions)**.**

**In your revised cover letter, please address the following prompts:**

**a) If there are ethical or legal restrictions on sharing a de-identified data set, please explain them in detail (e.g., data contain potentially identifying or sensitive patient information) and who has imposed them (e.g., an ethics committee). Please also provide contact information for a data access committee, ethics committee, or other institutional body to which data requests may be sent.**

We have shared the dataset.

**b) If there are no restrictions, please upload the minimal anonymized data set necessary to replicate your study findings as either Supporting Information files or to a stable, public repository and provide us with the relevant URLs, DOIs, or accession numbers. Please see** [**http://www.bmj.com/content/340/bmj.c181.long**](http://www.bmj.com/content/340/bmj.c181.long) **for guidelines on how to de-identify and prepare clinical data for publication. For a list of acceptable repositories, please see** [**http://journals.plos.org/plosone/s/data-availability#loc-recommended-repositories**](http://journals.plos.org/plosone/s/data-availability#loc-recommended-repositories)**.**

**We will update your Data Availability statement on your behalf to reflect the information you provide.**

We have uploaded the anonymized data set.

**REVIEWER #1**

**This paper uses cross sectional data on 2,000 married women 18 to 30 years of age selected from two low-med socioeconomic urban neighborhoods in Delhi, India to study health and nutrition related morbidities that are related to poor birth outcomes. The descriptive results show high rates of undernutrition, anemia, reproductive tract infections, hypothyroidism, and prediabetes. Multivariate results using logistic regression demonstrate associations of these morbidities to a basic set of variables such as age, education, and wealth.**

**On the whole, this is a well done study and I only have minor comments:
1. Women who did not give consent were excluded. It would be good to know the how many women were excluded and any information on how they may differ from the analysis sample.**

Of the women who had no other exclusion criteria, 13% did not consent to participate in the study. Some characteristics of the enrolled and non-enrolled women are given below.

**Baseline characteristics of enrolled and non-enrolled women**

| **Characteristics** | **Enrolled women**  **(n=2000)** | **Non-enrolled women**  **(n=869)** |
| --- | --- | --- |
| Height <150 cm, n (%) | 34.6% | 34.7% |
| Height (cm), Mean (SD) | 152.3 (5.6) | 151.7 (6.2) |
| Body Mass Index (kg/m^2^), Mean (SD) | 22.6 (4.3) | 22.9 (3.9) |
| Mid Upper Arm Circumference (cm), Mean (SD) | 25.7 (3.7) | 25.3 (3.1) |
| Women years of schooling, Median (IQR) | 10 (8, 13) | 10 (8, 12) |
| Total family income per year (INR), Median (IQR) | 200000 (144000, 264000) | 180000 (120000, 240000) |

**2. In the multivariate analysis, the authors only included baseline variables that had a p value less than .20 in bivariate analyses. This is typically not a good idea since the baseline variables are likely correlated and omitting variables could lead to biased effects for the included variables. A better strategy would be to use the complete set of baseline variables in all regressions.**

We have rerun the multivariable regression models using all the baseline variables namely women’s age, years of schooling, occupation, religion, household wealth quintile, family structure, women’s BMI. We have updated Table 4 accordingly. No major changes were observed in the effect size and its 95% CI. (Table 4; Pages 16-17).

**3. The coefficients in a logistic regression are scaled by an unknown factor. As a result, marginal effects which are not scale dependent are also often reported. Marginal effects are straightforward to calculate using the STATA statistical software that the authors used.**

We agree that the coefficients in a logistic regression are scaled by an unknown factor specifically for categorical variables. We have used the following command in STATA “**margins, dydx(*)**” to calculate the predicted probability of each baseline variable holding all the other explanatory variables constant. We have provided the predicted probability of each baseline variable with each morbidity (moderate to severe anemia, hypothyroidism, undernutrition, overweight or obesity, prediabetes or diabetes and symptoms and signs of RTI/STI) in the Supplement Table 1. The inference from logistic regression models and margins model is the same.

We have accordingly modified the Statistical analysis section (Lines 189-194).

**REVIEWER #2**

**Preconception health and nutrition are recognised to impact on maternal and birth outcomes and an understanding of the prevalence of maternal health conditions and sociodemographic correlates is therefore important and understudied in this setting. This is a generally well designed, conducted and written study.**

**Please perform a thorough proof-read to improve readability and punctuation. For example ‘planning special programs for the women’, ‘was such that it would require to be managed ‘ etc.**

Thank you this suggestion. We hope that the manuscript reads better now.

**There is some inconsistency in reporting of outcomes (eg variable inclusion or exclusion of overnutrition) and I would clarify this throughout the manuscript. The discussion is somewhat simplistic and would benefit from restructuring.**

We have now included overnutrition (overweight or obesity) as an outcome. We have also restructured the Discussion section and hope that it provides better clarity now.

**Line 23- I would reword the abstract of ‘We report prevalence of health and nutrition-related morbidities specifically, anemia, undernutrition, overweight and obesity, sexually transmitted infections (STI) or reproductive tract infections (RTI), diabetes or prediabetes, hypothyroidism, hypertension, and depressive symptoms during the preconception period, among women aged 18 to 30 years’ to health OR nutrition-related as I’m unclear how STI and RTI’s are nutrition related?**

Thank you. We have rephrased the sentence as “We report the prevalence of health or nutrition-related morbidities, specifically, anemia, undernutrition, overweight and obesity, sexually transmitted infections (STIs) or reproductive tract infections (RTIs), diabetes or prediabetes, hypothyroidism, hypertension, and depressive symptoms, during the preconception period among women aged 18 to 30 years” (Lines 63-67).

**Line 30 - As this is a subset of a randomised controlled trial, the generalisability of the population needs to be commented on as these women would have volunteered for recruitment to a much more intensive research study and hence would be assumed to be more motivated.**

We excluded women living in temporary housing as they are likely to be relocated by the government in the near future. However, height, BMI, MUAC, years of schooling, total family income per year between enrolled and excluded women were not very different (see the table on **Baseline characteristics of enrolled and non-enrolled women under Reviewer 1, Point 1).** We have commented on the generalizability of the population in the Discussion section (Lines 365).

**Line 39 - ‘Significant associations were observed for RTI/STI and undernutrition, hypothyroidism and diabetes or prediabetes with increasing age, low body mass index (BMI) and lower wealth quintiles. Anemia was inversely associated with women’s years of schooling.’ Please format appropriately to make the dependent and independent variables clearer.**

We have rephrased the statement (see below) to clearly indicate dependent and independent variables (Lines 40-45). We hope that the dependent and independent variables are clearer now.

“There was an increased risk of RTI/STI symptoms and signs in undernourished women and an increased risk of diabetes or prediabetes in overweight or obese women. An increased risk of undernutrition was also observed in women from lower categories of wealth quintiles. A decreased risk of moderate to severe anemia was seen in overweight women and those who completed at least secondary education.

**Line 53 – This is worded a little clumsily (eg ‘health-related morbidity’). Suggest rephrase**

We have now rephrased this as:

“Optimal health and nutritional status are essential for women of reproductive age. Early screening and treatment of morbidities are important to enable women to be prepared for future pregnancy**” (Lines 60-63).**

**Line 54-57 – I would provide further detail here about exactly what morbidities are associated with exactly what outcomes.**

We have provided details on the outcomes and the associated morbidities (Lines 66-70).

“Specifically, anemia has been associated with the likelihood of having a baby born with low birth weight (LBW); underweight has been associated with LBW and preterm birth; smoking has been associated with preterm birth; and hypothyroidism has been associated with preterm birth, intrauterine growth restriction and LBW.”

**Line 58-60 – I would distinguish here between the need for interventions during pregnancy to both manage under and over nutrition.**

We have specified that interventions during pregnancy that are needed to manage undernutrition (Lines71-74).

**Line 66 – ‘Reliable estimates of these morbidities in women of reproductive age are lacking in India.’ Is this the main research gap of this study? I would state this more clearly in the introduction and discussion**.

The main research gap in India is lack of reliable estimates of health or nutrition related morbidities specifically, anemia, undernutrition, overweight and obesity, sexually transmitted infections (STIs) or reproductive tract infections (RTIs), diabetes or prediabetes, hypothyroidism, hypertension, and depressive symptoms among women of reproductive age.

**Line 81 – Provide data to support this in the results (eg Table 1).**

We have provided the following information in the Results section (Lines 203-206).

The median (IQR) family income per year was 3333.3 (2500, 4300) USD, 90% of the households had bank accounts, and all enrolled households had concrete roofs and toilets, water connections within the house premises and legal electricity connections.

**Line 85 – Provide more detail on the signs and symptoms of STI/RTI assessed and how (eg clinical checklist based on what)?.**

We are following “National Guidelines on Prevention, Management and Control of Reproductive Tract Infections including Sexually Transmitted Infections”, Ministry of Health and Family Welfare, Government of India (<http://naco.gov.in/sites/default/files/National_Guidelines_on_PMC_of_RTI_Including_STI%201.pdf>) for assessing symptoms and signs of STI/RTI. The symptoms and signs of STI/RTI are described in the Definition section; these are also summarized below. (Lines 134-144).

“The symptoms of STIs/RTIs were assessed by trained study workers. An STI/RTI was considered if any one of the following symptoms were reported: swelling in the groin, dysuria, genital ulcer or sore, itching or burning sensation in the genital region, vaginal discharge, and pain in the lower abdomen. All women reporting one or more symptoms were examined by a physician, and an RTI was confirmed if any of the following signs were present: sores, blisters or ulcers in the genital area; foul-smelling, greenish or curdy white vaginal discharge; cervical erosion or mucopurulent pus at the cervical os; vaginal erythema with discharge; vulvar erythema, edema or induration; palpable lymph nodes in the inguinal area; and painful or palpable adnexa on bimanual examination, lower abdominal tenderness, or cervical motion tenderness [25]. Syphilis was diagnosed by the RPR test.”

**Line 86 – State if weight measured clothed/unclothed or fasting/non-fasting and if blood sample non-fasting.**

The weight was measured with clothes on and non-fasting blood sample was drawn. These have been specified in the manuscript (Line 104).

**Line 150 – Not all the morbidities mentioned in the abstract are in the sample size calculations (eg over nutrition). Please specify primary versus secondary outcomes (ideally a priori). Overnutrition is also mentioned in the discussion as an important morbidity despite lack of inclusion in these sample size calculations and regression models.**

We have now calculated the sample size for prevalence of overweight or obesity. Assuming, prevalence of overweight or obesity as ~25%, with 15% relative precision and 95% confidence level, 512 women of reproductive age group are required.

We did multivariable logistic regression to examine the association between baseline covariates (women’s age, education, occupation and BMI; religion of the head of the family; wealth quintiles; family structure) with overweight or obesity. We have now included these estimates in Table 4 (Pages 16-17).

**Line 159-160/Table 4- Again, not all outcomes are mentioned (eg over nutrition).**

We have now included overnutrition (overweight or obesity) in Table 4 (Pages 16-17).

**Line 161 – Please provide details of how the models were assessed for standard assumptions.**

The multivariable logistic regression models were assessed for independency, specification error (“linktest” command in STATA to calculate linear predicted value (_hat) and linear predicted value squared (_hatsq) , goodness-of-fit (we used Hosmer and Lemeshow’s goodness-of-fit test), multicollinearity ( “collin” command in STATA), influential observations ( “predict” command in STATA).

**Line 167 – Provide definition of stunting in the methods**.

We have provided the definition of stunting [height <150 cm (< -2 standard deviations of the World Health Organization standards; <https://www.who.int/childgrowth/en/>] in the Methods section (Lines 131-134).

**Line 188 – From this sentence it sounds like overweight/obesity don’t warrant medical treatment, is this the case?**

We have now included overweight in the definition of women with conditions that could be treated at primary level care and obesity in definition of women with any severe morbidity requiring specialized medical supervision and follow up. Accordingly, we have revised Table 2 (Table 2; Page 13).

**Provide data on the proportion of women who responded from the total eligible.**

Of the total eligible women who met the inclusion criteria, 3604 (85%) women were enrolled and 675 (13%) did not give consent.

**Figure 1 – Suggest provide in greyscale and 2D**

We have modified Figure 1 as requested.

**Table 4 – Not all significant results are reported in the text (e.g. BMI and anaemia), I would clearly state all significant relationships and then explain these in more** **detail.**

We have now reported all significant results in the text (Lines 250-261) and explained these in greater details in the Discussion section (Lines 292-293; Lines 300-302; and Lines 322-327).

**There are no line numbers in the discussion so it is difficult to make comments. The formatting of the discussion also makes it difficult to review as it’s difficult to see where new paragraphs sometimes commence. The discussion appears to be written in a way where there are numerous very short paragraphs without consistent description of the consistency of findings to prior research and potential mechanisms/implications of the findings. I would suggest restructuring to 5-6 larger paragraphs**

We have restructured the Discussion into 6 paragraphs and added line numbers.

**‘Animal studies have shown that undernutrition or deficiency of specific nutrients and
physiological status including hyperglycemia can affect the embryo with a potential for their future disease risk over their lifetime [2, 32-34]’ Please clarify mechanistically how different physiological states affect the embryo in different ways.**

We have now clarified the mechanisms how different physiological status affect the embryo in different ways (Line 352-360).
