# Supplementary material for: Burden of preconception morbidity in women of reproductive age from an urban setting in North India
Source: PLoS One. 2020 Jun 18;15(6):e0234768. doi: 10.1371/journal.pone.0234768 (PMC7302496; doi:10.1371/journal.pone.0234768)
Supplement: Attachment — Submitted filename: Responses to reviewers comments_21042020.docx [file pone.0234768.s005.docx]

**RESPONSES TO REVIEWERS COMMENTS**

**REVIEWER #3:**

**A cross-sectional study was conducted to report the prevalence of health or nutrition-related morbidities during the preconception period among women from North India. The study also aimed to predict health and nutrition-related outcomes. The prevalence of health and nutrition-related morbidities ranged from 10 to nearly 60%.

Minor revisions**

**1- Indicate the date range women participated in the study.**

The women were enrolled from July 1, 2017 to December 31, 2017 (Lines 86-87).

**2- Line 164: Provide a more comprehensive sample size calculation.
State and justify the study’s target sample size with a pre-study statistical power calculation. The power calculation should include: sample size, alpha level (indicating one or two-sided), minimal detectable difference and statistical testing method.**

Sample size calculations were based on confidence interval using single proportion with relative precision. We calculated sample size assuming a relative precision ranging between 10 and 30%, at 5% alpha level and assuming a non-response rate of 20% (Table below). These details have now been added under the section on sample size (Page 8). The table of sample sizes has been provided as Supplemental Table 1.

**Table 1: Sample sizes**

|  | **Severe anemia** | **Mild to Moderate anemia** | **BMI <18.5 kg/m^2^** | **BMI <16 kg/m^2^** | **BMI ≥25 kg/m^2^** | **Hypothyroid** | **STIs/RTIs** |
| --- | --- | --- | --- | --- | --- | --- | --- |
| Expected Prevalence (%) | 3 | 51 | 16 | 3 | 25 | 7 | 40 |
| Expected 95% Confidence Interval | 2-4 | 45-55 | 13-19 | 2-4 | 21-29 | 5-9 | 36-44 |
| Relative Precision (%) | 30 | 10 | 20 | 30 | 15 | 30 | 10 |
| Required sample size | 1380 | 369 | 504 | 1380 | 512 | 567 | 576 |
| Total sample size including 20% missing specimens | 1656 | 443 | 605 | 1656 | 620 | 680 | 691 |

Severe anemia: hemoglobin level <8 g/dL; Mild to Moderate anemia: hemoglobin level 8 to 11.9 g/dL; BMI <18.5 kg/m^2^: undernutrition; BMI <16 kg/m^2^: severe undernutrition; BMI ≥25 kg/m^2^: overweight or obesity; Hypothyroid: TSH levels >5.5 IU/mL or if TSH levels between 4.0 and 5.5 IU/mL and FT4 levels were <0.89 ng/dL; STI/RTIs: presence of both symptoms and signs

**3- Line 170: State the method used to estimate the 95% CIs.**

We used binomial exact method to estimate the 95% CIs.

**4- Table 1: Indicate when frequency (%) are represented**

Apologies, we have now indicated when frequencies (%) are represented (Page 10; Line 193)

**5- Line 203-8: Provide 95% confidence intervals associated with the incidence percentages.**

We have now provided 95% confidence intervals associated with the incidence percentages. (Pages 10-11)

**6- Table 3: Clarify that the 95% CIs are associated with the prevalence.**

We agree. The 95% CIs are associated with the prevalence.

**7- Include labeling that clearly identifies the morbidity factors.**

The following labeling was used for categorizing morbidities:

- Anemia: Reference category: No or mild anemia; other category: Moderate to severe anemia
- Hypothyroidism : Reference category: hypothyroidism absent ;other category: hypothyroidism present
- Undernutrition: Reference category: BMI ≥ 18.5 kg/m^2^ ;other category: BMI <18.5 kg/m^2^
- Overweight and obesity : Reference category: BMI < 25 kg/m^2^ ; other category: BMI ≥25 kg/m^2^
- Prediabetes or Diabetes : Reference category: HbA1c <5.7%; other category: HbA1c ≥5.7%
- Symptoms and Signs of STIs/RTIs : Reference category: women who had either symptoms and no signs present or no symptoms present ;other category: women who had both symptoms and signs present

The baseline variables were labelled as;

- Women’s age : continuous variable
- Women’s education : Reference category :None; category 1: primary (1-5 years);category 2: secondary (6-12 years);category 3: higher than secondary (>12 years)
- Women’s occupation : Reference category: working outside home ; other category :not working (housewife)
- Women’s BMI : Reference category: BMI 18.5 to 24.99 kg/m^2^ ;category 1: BMI <18.5 kg/m^2^; category 2: BMI 25 to 29.99 kg/m^2^ and category 3: BMI≥ 30 kg/m^2^
- Religion of the head of the family : Reference category :others (religions than Hindu );other category Hindu
- Wealth quintiles: Reference category: poorest; category 1: very poor; category 2: poor; category 3: less poor; category 4: least poor
- Family structure : Reference category :nuclear families; other category extended/joint families

**8- The method section indicates that "multivariable logistic regression models were assessed for independence of observations, specification error, goodness-of-fit, multicollinearity and influential observations." Provide a summary of these indicators for each of the multivariate logistic models. Include a general statement about the fit in the results section, and include the full details as supplemental material.**

We have described the fit of the models in the results section (Lines 248-253). This is reproduced below

All multivariable logistic regression models were assessed for specification error, goodness-of-fit, multicollinearity and influential observations. We did not find any significant specification error and influential observations for any of the multivariable logistic regression models. Hosmer and Lemeshow goodness-of-fit statistics showed that all models fit the data well. Variables included in the models did not show any collinearity. Details of the diagnostics for each model are provided in Supplemental Table 2.
